# Topical Cocaine Hydrochloride Nasal Solution: Anesthetic and Surgical Considerations

**DOI:** 10.7759/cureus.42804

**Published:** 2023-08-01

**Authors:** Salim C Lutfallah, Elise Brown, Noah J Spillers, Anamika Tandon, Rucha A Kelkar, Shahab Ahmadzadeh, Omar Viswanath, Giustino Varrassi, Sahar Shekoohi, Alan D Kaye

**Affiliations:** 1 School of Medicine, Louisiana State University Health Sciences Center at New Orleans, New Orleans, USA; 2 School of Medicine, Louisiana State University Health Sciences Center at Shreveport, Shreveport, USA; 3 School of Medicine, Medical University of South Carolina, Charleston, USA; 4 Anesthesiology, Louisiana State University Health Sciences Center, Shreveport, USA; 5 Pain Management, Valley Pain Consultants - Envision Physician Services, Phoenix, USA; 6 Pain Medicine, Paolo Procacci Foundation, Rome, ITA

**Keywords:** nasal surgery, topical solution, vasoconstriction, anesthetic, cocaine

## Abstract

Adequate surgical view during various types of nasal procedures is essential for surgical operations to be performed in a safe, efficient, and successful manner. Minimizing bleeding during surgery is an important way of increasing visualization that is commonly achieved by using a vasoconstrictive agent to control intraoperative hemorrhage. Many otolaryngologists choose to employ topical cocaine to minimize bleeding during surgery owing to its vasoconstrictive properties, while simultaneously benefitting from its dual local anesthetic effects. The relative benefit of topical cocaine for otolaryngologic procedures when compared to other topical analgesics and vasoconstrictors remains a topic of discussion due to the multiple potential cardiac and central nervous system side effects associated with cocaine administration. Furthermore, there is not a scientifically backed maximal safe dose published; instead, most of the guidelines for intranasal cocaine use are based on untested clinical practice. Despite this, the short latency, adequate duration of action, and inherent vasoconstrictive and decongestive capabilities make cocaine a valuable anesthetic agent for use in clinical procedures. As the relative benefit of using topical cocaine compared to the use of other vasoconstrictors and analgesics for nasal procedures remains undetermined in the current literature, this leaves the need for a comprehensive review of research that explores the risks and benefits of using topical cocaine in nasal procedures based on clinical trials that compare intranasal cocaine with various other analgesics and vasoconstrictors.

## Introduction and background

In 1884, the Austrian ophthalmologist Carl Koller described the tissue-numbing properties of cocaine by placing cocaine solution on the cornea and producing insensibility [1] Since then, the local anesthetic effects of the drug have continuously been evaluated. For over half a century, there were attempts to ban its use [2]; however, its unique pharmacological properties [3], such as its profound vasoconstriction, have made it a frequently used topical anesthetic for nasal surgery [4]. In January 2020, the Food and Drug Administration (FDA) approved cocaine nasal spray as a scheduled drug category II due to cocaine hydrochloride topical solution 4% as a topical anesthetic of the mucous membranes of the oral, laryngeal, and nasal cavities. However, no clear guidelines exist on utilizing cocaine hydrochloride to prepare the nose [5]. A study conducted among UK otorhinolaryngologists consisting of 360 consultant surgeons found that 66% used cocaine and adrenaline together for rhinological surgeries, and more than 40% used cocaine in pediatric patients [6]. A larger 2004 study including 4,717 members of the American Academy of Otolaryngology-Head and Neck Surgery found that 50% of respondents reported using cocaine as a topical agent during endoscopic sinus surgery in the preceding calendar year [7]. A maximal safe dose of 200 mg, or 1.5 mg/kg to 3 mg/kg, of intranasal cocaine is often used [8]; however, this dosage is based more so off untested clinical practice than rigorous scientific methods [9]. The inadequate guidelines for the use of cocaine as a nasal anesthetic have left a lot to be answered. In recent years, the safety and role of cocaine in nasal procedures have been questioned due to occasional case reports of adverse effects [10]. For this reason, a review of the literature is required to determine the indications and administration of cocaine as a topical solution compared to the possible adverse effects and contraindications. It is also essential for the efficacy of cocaine hydrochloride to be assessed against alternative analgesic methods commonly used in various nasal procedures.

## Review

Indications

Cocaine can be used for intranasal procedures to provide anesthesia to the surgical site while allowing vasoconstriction of nearby vessels. This reduces intraoperative bleeding and provides a clear view during endoscopic nasal procedures [11]. Cocaine hydrochloride nasal spray has been indicated as a topical anesthetic preparation for endoscopic nasal operative procedures and transnasal tube insertions such as nasotracheal and nasogastric tubes [12]. Topical cocaine has also been used in the outpatient clinical setting to perform rigid and flexible endoscopies in the office and used off-label as a temporary treatment for epistaxis before packing or cauterizing the site of bleeding [5,13]. While there are other anesthetics, such as lidocaine, that provide a similar profile, cocaine nasal spray may be favored by physicians during endoscopic procedures related to its inherent decongestant effect [14], its relatively low cost, and its intrinsic vasoconstrictive properties [13].

Administration

Cocaine nasal solution ranges from 4% to 10%, with 4% being the most frequently utilized [12]. Direct-application topical solutions are formulated for the mucosa as single-use 4 mL solutions or multiple-use 10 mL solutions. Each 1 mL of 4% solution contains 40 mg of cocaine hydrochloride [15]. A review of the literature recommends that to avoid systemic symptoms, the maximum safe dose is 1.5 mg/kg for adults, with most otolaryngologists using less than 200 mg [13]. As an anesthetic, cocaine is typically applied intranasally by inserting one to two cocaine-soaked cotton pledgets into each nasal cavity against the septum. The pledgets may be kept on the septum for up to 20 minutes. Once the pledgets are removed, the procedure may begin [16] or as a spray [13]. The topical nasal solution typically has a rapid onset of action with a duration lasting 30 to 60 minutes. An alternative administration method known as Moffett’s solution uses a mixture of 2 mL of 10% cocaine hydrochloride, 1 mL of 1:1,000 adrenaline, 2 mL of sodium bicarbonate, and 5 mL of sodium chloride. This method has been noted to increase hemostasis in the operative setting as well as to reduce cardiotoxicity [12,13,15]. Cocaine hydrochloride has also been utilized as a paste combined with liquid paraffin but is no longer readily administered due to its delayed rate of absorption [17].

Mechanism of action

The chemical structure of cocaine is a tropane alkaloid with weak basic properties. In its free form, cocaine is insoluble in aqueous solutions, but when ionized with hydrochloride salt, it becomes soluble in aqueous solutions [18]. Cocaine hydrochloride is generally the form in which cocaine nasal spray is applied due to its ability to be readily dissolvable in a mucus membrane such as the nasopharynx [18]. Cocaine used intranasally causes vasoconstriction of the vessels, which decreases the drug’s absorption. This has been noted to cause a 60-minute delay in peak concentrations, which should be accounted for when using cocaine nasal solution as preoperative anesthesia [18]. Cocaine nasal spray exerts local anesthetic effects by blocking voltage-gated sodium channels [11,18]. The inactivity of these sodium channels blocks the depolarization of the neuronal membrane and stops the propagation of action potentials [18]. Cocaine nasal spray also exerts sympathomimetic effects, inhibiting noradrenaline reuptake by blocking the noradrenaline transporter [18]. As reuptake of noradrenaline is inhibited, there is an increase in catecholamine availability, increasing the stimulation of alpha- and beta-adrenergic receptors. Stimulation of these receptors correlates to the marked vasoconstrictive properties that cocaine nasal spray can provide during endoscopic nasal procedures [18]. While the use of cocaine nasal spray has beneficial vasoconstrictive effects, it also influences the cardiovascular system by causing vasoconstriction of the coronary arteries, leading to tachycardia and hypertension that should be monitored with its use [14]. Cocaine nasal spray can also stimulate dopamine reuptake, leading to the activation of dopaminergic postsynaptic receptors, causing a euphoric effect following use in procedures; additionally, it can bind N-methyl-D-aspartate, sigma, and kappa opioid receptors [18]. Metabolism of cocaine nasal spray has been noted to follow first-order elimination, with an increase in drug concentration leading to an increased elimination rate [19]. Prior studies have estimated the overall half-life of cocaine nasal spray to be 70-80 minutes [18]. The liver metabolizes cocaine to form three metabolites: ecgonine methyl ester (EME) and benzoylecgonine (BE) are two inactive metabolites that are produced through hydrolysis in the liver, and the active metabolite norcocaine is formed by N-demethylation by cytochrome P450 via the enzyme CYP3A4. These three metabolites are renally excreted [18].

Adverse effects and toxicity

Cocaine is a popular street drug related to its propensity to get one high with a feeling of extreme euphoria. This effect is achieved by inhibiting the reuptake of the catecholamine dopamine from the synaptic cleft between axons [20]. This results in a feeling of euphoria, alertness, and confidence when cocaine is taken at lower doses. Conversely, when cocaine is used illicitly at higher doses, it can cause hallucinations, disorientation, and aggressiveness [20]. Cocaine also prevents the reuptake of other catecholamines such as norepinephrine and the monoamine neurotransmitter serotonin [21]. Increasing the amount of available norepinephrine in the synapse can also lead to central and peripheral vasculature vasoconstriction [22]. In the central nervous system, increasing the amount of serotonin by preventing its reuptake is thought to cause seizures as well as to play a role in the reward system of the addiction process in individuals who use cocaine illicitly [20,23]. Dating back to 1884, cocaine has been known to possess anesthetic properties and was even sold over the counter until 1916 [20]. Today, the use of cocaine is largely for its anesthetic properties as a local anesthetic for various procedures. Cocaine blocks voltage-gated sodium channels in neurons. Blockage of these ion channels prevents depolarization and subsequently inhibits the initiation and conduction of nerve impulses [20]. This mechanism by which anesthesia is produced is very similar to the rest of the local anesthetics. However, cocaine is the only local anesthetic that also produces behavioral responses, giving it an addictive potential [24].

Another potential toxicity occurs through cocaine’s ability to block sodium channels, thus classifying it as an antidysrhythmic drug [25]. Its slow on/off kinetics poses the potential to cause ventricular arrhythmias as well as a prolonged QT/QTc or wide QRS complex [20]. Additional cardiovascular adverse effects and toxicities can be seen when it comes to the vasoconstrictive effect cocaine has on peripheral vessels, as well as coronary vasoconstriction [26]. There have been many documented reports regarding cocaine-induced myocardial infarction (MI) and subsequent ischemia [27]. Following these reports, extensive research has detailed the risk associated with cocaine risk and MI. Following the administration of cocaine, individuals who are not even at risk of MI experience up to 24 times increased risk in 60 minutes. Importantly, these reports also state the subsequent risk of MI following cocaine use is independent of the amount used, the route of administration, and the frequency of its use [27]. Furthermore, a study testing the effect of cocaine on vasoconstriction using myocardial contrast echocardiography indicated that, in young people with no previous cocaine exposure, low-dose cocaine exposure showed sizeable decreases in myocardial perfusion [28]. In addition to MI, aortic dissection should also be considered when patients present with cocaine-associated chest pain. The incidence of aortic dissection is low and typically occurs in younger patients with pre-existing hypertension. Aortic dissection is thought to occur in these patients due to the sudden surge in blood pressure and tachycardia, placing enormous stress on the aorta itself [27].

While central nervous system and cardiovascular toxicities represent the most common adverse effects of cocaine use and abuse, there have also been adverse effects reported in various other systems, including pulmonary, musculoskeletal, and renal systems. In the pulmonary system, pneumothoraxes are thought to occur due to patient inhalation of cocaine; additionally, bronchoconstriction can occur post-administration, which can worsen asthma and asthmatic symptoms [27]. Musculoskeletal adverse effects include conditions such as ischemia from vasoconstriction and can potentially cause rhabdomyolysis. In a patient presenting with rhabdomyolysis symptoms, it is important to treat while pending results to prevent irreversible renal tubular damage due to the breakdown of hemoglobin and myoglobin products [27,29]. While cocaine’s anesthetic properties can certainly be useful, it is imperative that clinicians analyze the vast range of toxic effects this drug can cause, scrutinize the risk-to-reward ratio, and consider all options before administering cocaine to a patient.

Contraindications

Beta-blockers have been contraindicated in treating patients due to the potential adverse effects of vasoconstriction from cocaine combined with peripheral vasocontraction due to inhibiting the beta-adrenergic receptors. This would cause unopposed action of alpha-adrenergic mediated vasoconstriction, leading to increased hypertension and potential hypertensive crisis [20,26]. Cocaine and lidocaine both possess the same ability to block sodium channels. In an animal study, lidocaine exasperated cocaine-induced seizures. However, lidocaine’s kinetics are fast than cocaine’s and is thought to competitively bind the same receptors cocaine targets [20]. Class Ia and Ic antiarrhythmics are both contraindicated in cocaine administration due to the potential of additive sodium channel blockers increasing the risk of cocaine-induced QRS prolongation and/or arrhythmias [20]. Cocaine and succinylcholine are both metabolized by plasma cholinesterase. Using these drugs together can increase the toxicity of cocaine or succinylcholine and is contraindicated in therapeutic cocaine use [20]. Antidepressant drugs such as monoamine oxidase inhibitors prevent the breakdown of endogenous catecholamines and can be expected to have an additive effect on cocaine’s toxicity. Additionally, selective serotonin reuptake inhibitors have increased the incidence of cocaine-induced seizures [20]. In addition to all these prescription drugs, cocaine is also known to be an abused substance and can cause synergistic effects with other abused substances such as alcohol, nicotine, and marijuana. Therefore, it is important to discuss both prescription and over-the-counter medication use with the patient before utilizing cocaine in any way.

Clinical studies

The efficacy of cocaine nasal solution in the reduction of pain and various adverse effects secondary to otolaryngologic procedures has been studied vastly. One study compared the efficacy of various formulations of topical cocaine solutions in reducing nasotracheal intubation-induced epistaxis [30]. In this study, a 4% topical cocaine solution was compared to a 6% solution. The primary outcomes studied were the reduction in the incidence and severity of epistaxis during nasotracheal intubation, examined from the nasal cavity to the nasopharynx using a fiberoptic bronchoscope. Overall, no significant difference was found between the treatment groups. In the 4% treatment group, the incidence of epistaxis was found to be 43.6% (17/39), and in the 6% treatment group, the incidence was 50% (20/40). The grade of bleeding from the oropharyngeal space and the nostrils were also compared, and no difference was found. In both treatment groups, the primary site of bleeding was in the nasopharynx: 76.47% in the 4% treatment group, and 60% in the 6% treatment group. Another outcome that the study measured was the adverse hemodynamic effects of both treatment groups. In the 6% treatment group, the mean arterial pressure (MAP) and heart rate both increased significantly, while in the 4% treatment group, there was only a moderate increase in MAP and heart rate that was not significant. However, the hemodynamic profiles of both treatment groups did not have any significant effects on nasotracheal intubation. This study had a few limitations. Most notably, a placebo control was not implemented into the study design because a saline treatment is unethical to administer due to the absence of therapeutic efficacy. Another notable limitation was the exclusion of any patients who had difficult intubation or who faced anticipated difficulty. The team concluded that although both treatment groups had the same efficacy in preventing epistaxis due to nasotracheal intubation, the 4% topical cocaine solution is recommended due to fewer adverse hemodynamic effects and a lower chance of toxicity.

Another study compared the anesthetic and vasoconstrictive efficacy of intranasal cocaine with xylometazoline/lidocaine solution [31]. This study’s primary outcomes were pulse, systolic blood pressure, diastolic blood pressure, nasal cross-sectional area, and pin-prick sensation. In the cocaine treatment group, the change in pulse pre-administration versus post-administration was insignificant (p > 0.05). Similarly, in the xylometazoline/lidocaine treatment group, the pulse change was also insignificant (p > 0.05). The change in systolic blood pressure for both treatment groups was also not statistically significant; however, the change in diastolic blood pressure for the cocaine treatment group was statistically significant with a value of +3.25 and a p-value of 0.041. Examining the change in pin-prick sensation between both treatment groups, the average change in sensation between the pre- and post-treatment within the cocaine group was relatively small (-1.85) and proved to be statistically significant with a p-value of 0.0001. In the xylometazoline/lidocaine treatment group, the difference in pin-prick sensation was also small (-0.90) and was significant with a p-value of 0.038. An analysis of variance was done on the post-condition values of both treatment groups, and no significant difference in anesthesia was determined between the cocaine group and the xylometazoline/lidocaine group (p > 0.05). Overall, comparing the effect of cocaine with xylometazoline/lidocaine on hemodynamic and anesthetic variables, both treatment groups had comparable efficacy. Both treatment groups provided a minor anesthetic effect, with no significant difference found between them. Regarding hemodynamic variables, the only notable difference between the two groups was the increase in diastolic blood pressure in the cocaine treatment group.

The effects of cocaine versus tetracaine/adrenaline on producing local anesthesia during septoplasty were explored in a 2006 study [32]. A total of 114 subjects were selected from patient groups who had a nasal septum deviation and received septoplasty with local anesthesia. Exclusion criteria consisted of those who were on anxiolytic, hypnotic, or antidepressant medications. Selected subjects were randomly divided into two treatment groups, namely, groups A and B. Group A was treated with 5 mL of 4% cocaine solution and group B was treated with 5 mL of 2% tetracaine solution + adrenaline. The final analysis included 108 out of the original 114 selected. The primary outcome measured was local anesthetic effects and was measured using the visual analog pain scale, which is a validated, subjective scale to measure acute and chronic pain. The scaling goes from 1 to 10, with 1 being “no pain” and 10 being “the worst pain.” The data from this visual analog scale were analyzed using the Mann-Whitney U test and in the context of each case. The average score of group A treated with cocaine solution was 4.46 with a standard deviation of 2.08. The average score of group B treated with tetracaine-adrenaline was 3.06 with a standard deviation of 1.47. Overall, although both treatment groups were able to reduce pain sensation following septoplasty, tetracaine was superior in its anesthetic profile, as well as its adverse effect profile. This study supported the use of tetracaine over cocaine for anesthesia and recommended against the use of cocaine (Table 1).

**Table 1:** Comparative studies.

Author (year)	Groups studied and intervention	Results and findings	Conclusions
Study 1: Lu et al. [30]	A 2014 double-blind experimental study measured the effects of 4% versus 6% topical cocaine nasal solution on the reduction of epistaxis in patients who underwent nasotracheal intubation	The study found that the incidence of epistaxis in the 4% cocaine solution was 43.6% (17/39) and 50% (20/40) in the 6% treatment group. The study also found that in the 6% treatment group, mean arterial pressure and heart rate were increased significantly, whereas the 4% treatment group only experienced a moderate increase in these variables that was not significant	Overall, the data suggest that the 4% topical cocaine solution is recommended for the treatment of nasotracheal intubation-induced epistaxis due to fewer adverse hemodynamic effects and a lower chance of toxicity
Study 2: Campbell et al. [31]	A 1992 randomized, double-blinded, placebo-controlled study investigated the anesthetic and hemodynamic effects of intranasal cocaine versus intranasal xylometazoline/lidocaine in healthy patients	The study found that intranasal cocaine significantly increased diastolic blood pressure. The study also found that the anesthetic effects of both treatment groups were relatively small, and when an analysis of variance was conducted, no significant difference was found between both groups	Overall, the data suggest that intranasal xylometazoline/lidocaine solution is preferred in the treatment of intranasal anesthesia compared to intranasal cocaine due to a lower risk of adverse hemodynamic outcomes
Study 3: Drivas et al. [32]	A longitudinal prospective, randomized, controlled trial examined patients who underwent septoplasty from December 2002 to February 2005 to evaluate the efficacy of 4% cocaine solution versus 2% tetracaine + adrenaline solution in inducing local anesthesia during septoplasty	The study found that subjects given tetracaine experienced significantly less pain compared to subjects who were given cocaine	Overall, the data suggest that tetracaine should be the first choice of anesthetic for nasal septoplasty and the use of cocaine should be avoided
Study 4: McGrath et al. [33]	A phase III, randomized, prospective, double-blind, multicenter, single-dose, placebo- and dose-controlled, parallel-group study investigated the safety and efficacy profile of 4% and 8% solutions of cocaine as topical anesthetic solutions in patients undergoing otolaryngology procedures	The study found that both the 4% and 8% cocaine solution significantly induced local anesthesia. Compared to the placebo group (9.5%), the 4% treatment group was able to increase the percentage of patients who achieved analgesia by 81.1% (p < 0.0001). The 8% treatment group significantly increased the percentage of patients (77.3%; p < 0.0001) when compared to the placebo	Overall, the study found that both the 4% and 8% cocaine solution produced significant analgesia in patients when compared to the placebo. The study recommends the use of topical cocaine solutions for local anesthesia in diagnostic procedures and surgeries on or through the nasal mucus membranes
Study 5: Cara et al. [34]	A 2003 randomized cross-over study evaluated the anesthetic efficacy of 5% cocaine solution versus 5% co-phenylcaine Forte solution applied during nasal intubation in healthy patients	The study found that there was no significant difference in pain perception during nasal intubation between the treatment groups	Overall, the study suggests that the use of co-phenylcaine should be used over cocaine due to the higher risk of adverse cardiovascular and vasoconstrictive effects associated with cocaine

## Conclusions

Cocaine has many innate properties, making it a valuable option for otolaryngologic procedures. Among these properties is its ability to act as a local anesthetic, vasoconstrictor, and decongestant with a short latency and adequate duration of action at a relatively low cost. However, there are many adverse effects associated with cocaine use that influence the cardiac, central nervous, pulmonary, musculoskeletal, and renal systems. Multiple studies have been conducted to determine the risk/benefit of the use of cocaine with variable results. When comparing various cocaine solutions (e.g., 4%, 6%, 8%), it was shown that all concentrations were effective analgesics, with 4% holding preference due to its lower rate of hemodynamic and toxic adverse effects. However, when comparing cocaine to other analgesics such as xylometazoline/lidocaine, tetracaine, and co-phenylalanine, it was found that alternative analgesics take preference due to decreased risk of various cardiovascular adversities. Cocaine remains a viable option for otolaryngologists who wish to perform nasal procedures with localized anesthesia, but alternative choices may prove to be better options related to decreased risk profile and similar effects.

## References

[1] Goerig M, Bacon D, van Zundert A (2012). Carl Koller, cocaine, and local anesthesia: some less known and forgotten facts. Reg Anesth Pain Med.

[2] Schenck NL (1975). Local anesthesia in otolaryngology. A re-evaluation. Ann Otol Rhinol Laryngol.

[3] Richards JR, Laurin EG (2023). Cocaine. http://www.ncbi.nlm.nih.gov/books/NBK430769/.

[4] Alhaddad ST, Khanna AK, Mascha EJ, Abdelmalak BB (2013). Phenylephrine as an alternative to cocaine for nasal vasoconstriction before nasal surgery: a randomised trial. Indian J Anaesth.

[5] Saif AM, Farboud A, Delfosse E, Pope L, Adke M (2016). Assessing the safety and efficacy of drugs used in preparing the nose for diagnostic and therapeutic procedures: a systematic review. Clin Otolaryngol.

[6] De R, Uppal HS, Shehab ZP, Hilger AW, Wilson PS, Courteney-Harris R (2003). Current practices of cocaine administration by UK otorhinolaryngologists. J Laryngol Otol.

[7] Dwyer C, Sowerby L, Rotenberg BW (2016). Is cocaine a safe topical agent for use during endoscopic sinus surgery?. Laryngoscope.

[8] Kubo N, Nakamura A, Yamashita T (1995). [Efficacy and complications of topical cocaine anesthesia in functional endoscopic sinus surgery]. Nihon Jibiinkoka Gakkai Kaiho.

[9] Long H, Greller H, Mercurio-Zappala M, Nelson LS, Hoffman RS (2004). Medicinal use of cocaine: a shifting paradigm over 25 years. Laryngoscope.

[10] Kara CO, Kaftan A, Atalay H, Pinar HS, Oğmen G (2001). Cardiovascular safety of cocaine anaesthesia in the presence of adrenaline during septal surgery. J Otolaryngol.

[11] Noorily AD, Noorily SH, Otto RA (1995). Cocaine, lidocaine, tetracaine: which is best for topical nasal anesthesia?. Anesth Analg.

[12] Valdes CJ, Bogado M, Rammal A, Samaha M, Tewfik MA (2014). Topical cocaine vs adrenaline in endoscopic sinus surgery: a blinded randomized controlled study. Int Forum Allergy Rhinol.

[13] Harper SJ, Jones NS (2006). Cocaine: what role does it have in current ENT practice? A review of the current literature. J Laryngol Otol.

[14] Smith JC, Rockley TJ (2002). A comparison of cocaine and 'co-phenylcaine' local anaesthesia in flexible nasendoscopy. Clin Otolaryngol Allied Sci.

[15] Lips FJ, O'Reilly J, Close D, Beaumont GD, Clapham M (1987). The effects of formulation and addition of adrenaline to cocaine for haemostasis in intranasal surgery. Anaesth Intensive Care.

[16] Liao BS, Hilsinger RL Jr, Rasgon BM, Matsuoka K, Adour KK (1999). A preliminary study of cocaine absorption from the nasal mucosa. Laryngoscope.

[17] Quiney RE (1986). Intranasal topical cocaine: Moffett's method or topical cocaine paste?. J Laryngol Otol.

[18] Roque Bravo R, Faria AC, Brito-da-Costa AM (2022). Cocaine: an updated overview on chemistry, detection, biokinetics, and pharmacotoxicological aspects including abuse pattern. Toxins (Basel).

[19] Wilkinson P, Van Dyke C, Jatlow P, Barash P, Byck R (1980). Intranasal and oral cocaine kinetics. Clin Pharmacol Ther.

[20] Goldstein RA, DesLauriers C, Burda AM (2009). Cocaine: history, social implications, and toxicity--a review. Dis Mon.

[21] Yuen J, Goyal A, Rusheen AE (2022). Cocaine increases stimulation-evoked serotonin efflux in the nucleus accumbens. J Neurophysiol.

[22] Goldfrank LR, Hoffman RS (1991). The cardiovascular effects of cocaine. Ann Emerg Med.

[23] Lasoń W (2001). Neurochemical and pharmacological aspects of cocaine-induced seizures. Pol J Pharmacol.

[24] Kreek MJ, Levran O, Reed B, Schlussman SD, Zhou Y, Butelman ER (2012). Opiate addiction and cocaine addiction: underlying molecular neurobiology and genetics. J Clin Invest.

[25] Kerns W 2nd, Garvey L, Owens J (1997). Cocaine-induced wide complex dysrhythmia. J Emerg Med.

[26] Zimmerman JL (2012). Cocaine intoxication. Crit Care Clin.

[27] Lange RA, Hillis LD (2001). Cardiovascular complications of cocaine use. N Engl J Med.

[28] Gurudevan SV, Nelson MD, Rader F (2013). Cocaine-induced vasoconstriction in the human coronary microcirculation: new evidence from myocardial contrast echocardiography. Circulation.

[29] Bosch X, Poch E, Grau JM (2009). Rhabdomyolysis and acute kidney injury. N Engl J Med.

[30] Lu IC, Hsieh YH, Hsu HT, Chen CH, Hsu CW, Tseng KY, Cheng KI (2014). Comparison of 4% and 6% topical cocaine solutions for reduction of epistaxis induced by nasotracheal intubation. Acta Anaesthesiol Taiwan.

[31] Campbell JP, Campbell CD, Warren DW, Prazma TU, Pillsbury HC 3rd (1992). Comparison of the vasoconstrictive and anesthetic effects of intranasally applied cocaine vs. xylometazoline/lidocaine solution. Otolaryngol Head Neck Surg.

[32] Drivas EI, Hajiioannou JK, Lachanas VA, Bizaki AJ, Kyrmizakis DE, Bizakis JG (2007). Cocaine versus tetracaine in septoplasty: a prospective, randomized, controlled trial. J Laryngol Otol.

[33] McGrath J, McGrath A, Burdett J, Shokri T, Cohn JE (2020). Investigation of topical intranasal cocaine for sinonasal procedures: a randomized, phase III clinical trial. Int Forum Allergy Rhinol.

[34] Cara DM, Norris AM, Neale LJ (2003). Pain during awake nasal intubation after topical cocaine or phenylephrine/lidocaine spray. Anaesthesia.

